# *C. albicans UME7* deletion does not have major impacts on white opaque switching, filamentation, or virulence

**DOI:** 10.17912/micropub.biology.000826

**Published:** 2023-05-25

**Authors:** Ben Evans, Evan Spell, Douglas Bernstein

**Affiliations:** 1 Biology, Ball State University, Muncie, Indiana, United States

## Abstract

*C. albicans* is the most prevalent human fungal pathogen, and can be especially dangerous to immunocompromised individuals. One key aspect of *C. albicans* virulence is morphological plasticity. *C. albicans* can undergo a number of distinct morphological changes and these changes are controlled by complex transcriptional networks. The transcription factor Ume6 is an important member of these networks, playing an essential role mediating filamentation. *C. albicans* , however encodes a second *UME6* homolog, *UME7* . *UME7* is highly conserved in the CTG fungal clade, but the role of *UME7* in *C. albicans* biology is unknown. Here we truncate and delete *C. albicans UME7* . We find Ume7 is dispensable for growth and filamentation. We also find that deletion does not have major consequences on virulence or white opaque switching. Our results suggest that under standard laboratory conditions deletion of *UME7* does not have large effects on *C. albicans* phenotype leaving its role in *C. albicans* biology undefined.

**Figure 1. Domain structure, growth, filamentation, virulence, and white opaque switching in wild type C. albicans and UME7 mutant.**
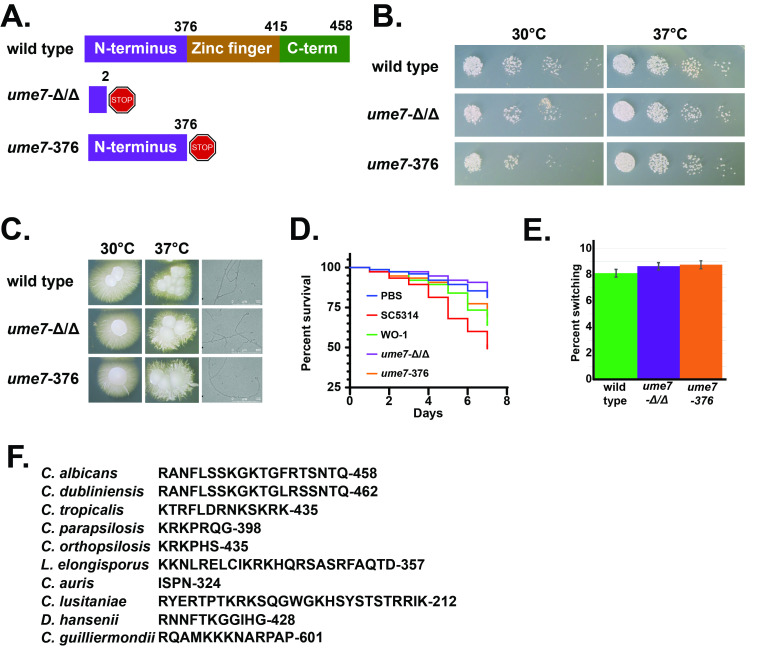
**A.** Domain structure of wild type *C. albicans* Ume7 and truncation mutants used in this study. **B.** Comparison of wild type, *ume7* Δ/Δ, and *ume7-736* growth at 30 and 37 C° on YPD. **C.** Comparison of filamentation between wild type, *ume7* Δ/Δ, and *ume7-736* at 30 and 37 C° on Spider media *.* **D.** Comparison of virulence in a *Galleria mellonella* infection model between wild type, *ume7* Δ/Δ, and *ume7-736.* Data were assessed via Kaplan Meier survival analysis and by the log rank test for multiple comparisons. **E.** Comparison of white to opaque switching rates between wild type, *ume7* Δ/Δ, and *ume7-736* **F.** Sequence alignment of Ume7 C-terminal tail domain.

## Description

*C. albicans* is the most prevalent human fungal pathogen asymptomatically colonizing gut mucosal tissue (Beck-Sague and Jarvis 1993). While colonization is usually harmless, in immunocompromised individuals it can lead to opportunistic *C. albicans* infections (Kim and Sudbery 2011) . *C. albicans* can infect a wide range of host tissues and infections range in severity from irritating mucosal infections, such as thrush, to life threatening systemic candidemia (Pfaller and Diekema 2010) . Invasive candidiasis in immunocompromised individuals has an estimated mortality up to 40% (Wey et al. 1988) (Viudes et al. 2002) . Few treatment options are available for systemic *C. albicans* infection and the identification of drug resistant isolates has increased the need to further understand the biology of this fungal pathogen.

In response to stress, *C. albicans* like many human fungal pathogens, can undergo morphological changes. One such change *C. albicans* can undergo is white to opaque switching (Slutsky et al. 1987). White cells are the more common morphology, but stress can induce cells to switch to the opaque morphology in which the cells are more elongated and oval (Alby and Bennett 2009) . Opaque cells will grow at 25 C°, but switch to the white morphology when shifted to 37 C° (Rikkerink, Magee, and Magee 1988) . White opaque switching is controlled by a complex transcriptional network (Hernday et al. 2013) . There is some evidence to suggest opaque cells are important for pathogenesis (Solis et al. 2018) , but the exact role for switching in pathogenesis is not well understood.

One of the most notable aspects of opaque cell biology, is that they mate much more frequently than white cells (Miller and Johnson 2002) . *C. albicans* mating requires cells of opposite mating type fuse and mix genetic material. After this, *C. albicans* loses chromosomes until it reaches a diploid state (Bennett and Johnson 2005) . Even though *C. albicans* encodes homologs for many genes known to be important for meiosis in other fungal species, meiosis in *C. albicans* has not been observed. Two such homologs are Ume6 and Ume7. In *S. cerevisiae,* Ume6 is important for meiosis progression (Strich et al. 1994) and Ume6 has been shown to play important roles in *C. albicans* filamentation (Banerjee et al. 2008) . The Ume6 family of transcription factors contain a conserved domain structure consisting of a N-terminal domain, Zinc-finger domain, and C-terminal domain. The Zinc-finger domain more highly conserved than the N-terminal or C-terminal domains (Skrzypek et al. 2017) . The Zinc-finger domain is thought to bind DNA and in our companion manuscript we present evidence that the C-terminal domain supports this function. Both Ume6 and Ume7 are highly conserved throughout the CTG fungal clade, but as of yet, the roles of Ume7 have not been determined (Skrzypek et al. 2017) .

We used CRISPR mediated genome editing to make two *UME7* mutants in the White Opaque (WO-1) background. The WO-1 strain background has been used to study white opaque switching previously (Slutsky et al. 1987). The first mutant *ume7* Δ/Δ replaced the second codon of *UME7* with a stop codon, making a complete functional deletion **Figure 1A** . The second strain we generated, inserted a stop codon at codon 376, *ume7-376* **Figure 1A** *.* This mutation generates a protein lacking the Zinc-finger and C-terminal tail domains. Next, we tested if these mutant strains grew more slowly than wild type. We found both *ume7* Δ/Δ and *ume7-736* grew at the same rate as wild type, and we did not detect changes in filamentation on solid media for either strain **Figure 1 B and C** . We also tested for differences in filamentation in liquid media and did not observe a change in phenotype. We then tested if either *ume7* Δ/Δ or *ume7-736* had a defect in virulence using a *G. mellonella* infection assay. It is possible the *ume7* Δ/Δ had a modest defect in virulence, P value 0.0052, **Figure 1 D** but we hesitate to place great biological significance on this finding as WO-1 is less virulent than clinical isolates such as SC5314, P value 0.0515, and the difference between WO-1 and *ume7* Δ/Δ is modest **Figure 1 D** . Differences between virulence for WO-1 and ume7-376 were not found to be statistically significant, P value 0.5235. Ume7’s homolog in *S. cerevisiae* is important for meiosis (Strich et al. 1994) but *C. albicans* does not undergo meiosis. White opaque switching is important however for *C. albicans* mating and we hypothesized that *C. albicans* *UME7* could be important for white opaque switching. We tested if *ume7* Δ/Δ or *ume7-736* switched at a different rate than wild type and did not find a significant difference in rate between wild type and mutant strains **Figure 1 E** .

We were unable to identify any obvious phenotypes that could be attributed to deletion or truncation of *C. albicans* *UME7* and previous work by others has noted that biofilm formation does not change in the *UME7* mutants (Nobile and Mitchell 2005) . Interestingly, *UME7* is conserved throughout the CTG clade (Skrzypek et al. 2017) . Furthermore, when we examined the Alphafold predictions for Ume7, they share many characteristics with those of Ume6. The N-terminal domain of both proteins are predicted to be either labile or unstructured and the Zinc-finger domains are predicted to form a classical Zinc-finger fold. The far C-termini of both proteins are predicted to form a long alpha helix in all species for which predictions are given. The sequence of these C-terminal domains is not conserved between species or between Ume6 and Ume7 **Figure 1 F** . We recently identified a Asn residue sidechain in the C-terminal alpha helix of *C. albicans* Ume6 that potentially helps coordinate its Zinc-finger domain. We identified a similar interaction in the Alphafold prediction of *C. albicans* Ume7 but this residue and the interaction were not conserved in the Alphafold prediction of Ume7 homologs.

Our data does not suggest an obvious function for *C. albicans* Ume7. As Ume7 is a putative transcription factor, RNAseq comparing mutant and wild type strain gene expression could potentially be used to help focus a search for a phenotype. However, *C. albicans* encodes 70 Zinc-finger transcription factors which could potentially confound interpretation of such results (Maicas et al. 2005) .

## Methods

***C. albicans* Strain Generation:** Vectors pV1093 were linearized via digestion with KpnI and SacI. Guide RNA primers targeting *UME7* were cloned into linearized vector. Repair templates encoding premature stop codons or point mutations in *UME7* were synthesized. Repair templates and linearized vector were co-transformed into *C. albicans* WO-1 via lithium acetate transformation. Transformations were plated on YPD supplemented with 200 µg/ml Nat for 3 days at 30°C. Single colonies were isolated on YPD supplemented with 100 µg/ml Nat and grown for 3 days at 30°C. Mutants were identified via restriction digest screening and DNA sequencing

**Growth and Filamentation:** Wild type, *ume7Δ/Δ* , and *ume7-736* were grown overnight in YPD at 25°C. Each strain was diluted to 0.1 OD _600_ . 4-fold serial dilutions were plated on YPD and Spider media. Plates were incubated at 37°C. Images were taken from 24 hours to 9 days. For liquid filamentation assays, strains were diluted to 0.1 OD _600_ in Spider media and were incubated at 37°C for 24 hours.

**White/opaque switching:** Wild type, *ume7Δ/Δ* , and *ume7-376* were grown on YPD plates at 30°C overnight. Single colonies were isolated on Lee’s for 5 days at 25°C. To obtain uniformly opaque colonies, single colonies were re-isolated on Lee’s for 5 days at 25°C. Single opaque colonies were suspended in Lee’s media. 100 cfu were plated on each of 6 YPD/0.1% glucose plates containing 5 µg/ml phloxine B. Plates were parafilmed and incubated at 25°C for 4 days. Switching frequency was determined as the total number of opaque and opaque-sectored colonies divided by the total number of colonies. Nine replicates of each strain were tested.

***Galleria mellonella* virulence:** Healthy larvae were assigned to sterile PBS, wild type, or Ume7 mutant injections. Strains were grown overnight at 37°C in YPD. Strains were washed twice with sterile PBS and resuspended in PBS at 10 ^6^ cells/mL. A Hamilton syringe was sterilized 70% ethanol and rinsed with PBS prior to each injection. Larvae were swabbed with 70% ethanol. 10 µl of PBS or 10 µl of cell suspension were injected into a rear proleg. Larvae were incubated in glass dishes at 37°C. Survival was assessed daily by visual inspection and prodding with a sterile pipet tip. Deceased larvae were removed daily.

## Extended Data

Description: raw data for white opaque switching. Resource Type: Dataset. DOI: 10.22002/p0shh-48m13
